# Brain–computer interface: trend, challenges, and threats

**DOI:** 10.1186/s40708-023-00199-3

**Published:** 2023-08-04

**Authors:** Baraka Maiseli, Abdi T. Abdalla, Libe V. Massawe, Mercy Mbise, Khadija Mkocha, Nassor Ally Nassor, Moses Ismail, James Michael, Samwel Kimambo

**Affiliations:** 1 Department of Electronics and Telecommunications Engineering, College of Information and Communication Technologies, University of Dar es Salaam, 14113 Dar es Salaam, Tanzania; 2 Department of Computer Science and Engineering, College of Information and Communication Technologies, University of Dar es Salaam, 14113 Dar es Salaam, Tanzania

**Keywords:** Brain–computer interface, Brain activity, Machine learning, Neurological disease, Signal processing, Augmented reality

## Abstract

Brain–computer interface (BCI), an emerging technology that facilitates communication between brain and computer, has attracted a great deal of research in recent years. Researchers provide experimental results demonstrating that BCI can restore the capabilities of physically challenged people, hence improving the quality of their lives. BCI has revolutionized and positively impacted several industries, including entertainment and gaming, automation and control, education, neuromarketing, and neuroergonomics. Notwithstanding its broad range of applications, the global trend of BCI remains lightly discussed in the literature. Understanding the trend may inform researchers and practitioners on the direction of the field, and on where they should invest their efforts more. Noting this significance, we have analyzed 25,336 metadata of BCI publications from Scopus to determine advancement of the field. The analysis shows an exponential growth of BCI publications in China from 2019 onwards, exceeding those from the United States that started to decline during the same period. Implications and reasons for this trend are discussed. Furthermore, we have extensively discussed challenges and threats limiting exploitation of BCI capabilities. A typical BCI architecture is hypothesized to address two prominent BCI threats, privacy and security, as an attempt to make the technology commercially viable to the society.

## Introduction

Naturally, humans use their peripheral nerves and muscles to interact with the outside physical environments in carrying out the desired actions. This necessity and premise for survival comes with a cost for people with severe neurological diseases, including amyotrophic lateral sclerosis and brainstem stroke. These people cannot control external devices, thus requiring assistance from healthy people that may not always be available. Challenged by the limitation, scientists and researchers have developed a brain–computer interface (BCI) technology that can transform brain signals into human actions independent of the peripheral nerves or muscles.

BCI, also called brain–machine interface, provides direct communication between brain and external devices, such as computers and robotic limbs [1–4]. Bypassing the conventional communication channels for different tasks (e.g., vision, movement, and speech), BCI links the brain’s electrical activity and the external world to augment human capabilities in interacting with the physical environment [1]. BCI provides a non-muscular communication channel and facilitates acquisition, manipulation, analysis, and translation of brain signals to control external devices or applications.

Since its conception in 1973 by Vidal [5], BCI has remained an active area of research with enormous promising opportunities [6–14]. Researchers have, for instance, reported remarkable achievements demonstrating that BCI can efficiently restore capabilities of people with disabilities, such as those with schizophrenia symptoms (psychosis, emotional disturbances, and cognitive dysfunction) [15–21]. Generally, BCI applications can be classified depending on the industry: gaming and entertainment [22–24], security and authentication [25], healthcare [21], education [26–28], advertisement and neuromarketing (commercial marketing using principles of neuroscience and cognitive science) [29–33], and neuroergonomics (application of neuroscience to ergonomics) [34, 35]. Given its cross-cutting nature across many aspects of developments, BCI may remain an attractive and a competitive research area over a longer period.

Despite the promising applications of BCI, there has been a paucity of studies on the future of this technology and its possible threats when applied to humans. The present study covers typical BCI threats, including medical safety, privacy, ethics, and security. We stimulate discussions within the scholarly community on the readiness to adopt the BCI technology and accommodate its challenges and potential threats. Furthermore, because the natural working principles of the brain are not comprehensively understood, recommendations have been provided for researchers to focus more on the short- and long-term impacts of BCI on the general welfare of humans. In addition, our study surfaces several research opportunities in the field of brain–computer interface. Researchers and practitioners may capitalize on these opportunities to develop safe BCI products that advance humanity and improve quality of our lives.

Lastly, we extracted 25,336 metadata from Scopus to analyze patterns and trend of BCI research. Results show an exponential growth of BCI publications, China being the leading country between 2019 onwards followed by the United States within the same period. This observation signals the significance of BCI to the community, but raises critical questions on the potential BCI threats to humans.

## Fundamental components of BCI system

**Fig. 1:**
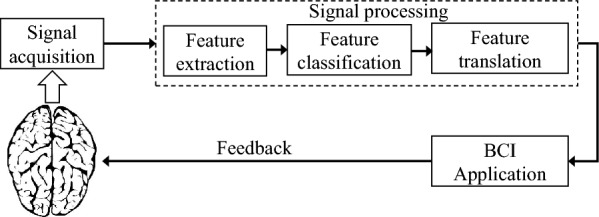
Main components of the brain–computer interface (BCI) system

The BCI system comprises three fundamental components that serve specific roles: signal acquisition, signal processing, and application (Fig. 1). These components are interconnected and work together to allow the flow of brain signals to the target BCI application (e.g., robotic arm). In particular situations, control signals from the BCI application may be sent back to the brain to stimulate some common human functionalities, such as vision and hearing.

### Signal acquisition

This component comprises an electronic device with electrodes for acquiring brain signals (oscillating electrical voltages caused by biological activities of the brain) that define its neurophysiological states. Signal acquisition involves capturing of electrophysiological signals that represent specific activities of the brain (e.g., movement, speech, hearing, and vision). Most BCI systems, including the commercial ones, deal with the following electrophysiological signals: electroencephalography, brain’s electrical activity measured with electrodes placed on the scalp [36, 37]; electrocorticography [38–40], electroencephalographic signals measured directly with electrodes placed on the surgically exposed cerebral cortex; local field potential [41], electric potential measured around the neuron’s extracellular space; and neuronal action potential [42, 43], rapid and temporary change in the neuron’s membrane potential. Before being presented to the next BCI component, the captured brain signals undergo filtering, amplification, and digitization [21]. The overall performance of the BCI system depends heavily on the quality (signal-to-noise ratio) of the acquired brain signals.

Depending on the signal acquisition method, BCI can broadly be categorized into two types: invasive (electrodes implanted under the scalp to record signals directly from the brain) and non-invasive (electrodes implanted on the scalp). Invasive BCI provides a more accurate reading of brain signals, but requires surgery; non-invasive BCI does not require surgery, but suffers from weak brain signals (poor signal-to-noise ratio) that require expensive amplification hardware and sophisticated signal processing techniques.

### Signal processing

#### Feature extraction

In this stage, the BCI system extracts critical electrophysiological features from the acquired signals to define brain activities, and hence encoding of the user’s intent [21]. Similar to the previous stage, feature extraction should be executed accurately, ensuring that the features reflect high correlation with the user’s intent to enhance the effectiveness and performance of the BCI system. Typical BCI systems employ time-domain or frequency-domain features [44–51] that take different characteristics: amplitude or latency of event-evoked potentials (e.g., P300), frequency power spectra (e.g., sensorimotor rhythms), or neuronal firing rates [21]. Therefore, before designing the BCI system, the domain transform and characteristics of features should be established. Also, confounding artifacts contained in the features that can negatively impact the subsequent stages of the BCI system should be eliminated.

#### Feature classification

The extracted features represent brain activities intended for desired actions. The classification process helps to recognize patterns of the features corresponding to these actions. For example, we can recognize features representing an instruction for moving a robotic arm. This component is usually implemented using machine learning and classification methods [52–54].

#### Feature translation

In this signal processing stage, the classified features are translated and transformed into actual commands to operate an external device (BCI application). Examples of the outputs given after feature extraction include commands for cursor movement on the computer screen, volume control on the audio device, or text writing. One important attribute of an algorithm for feature translation is adaptability [55, 56]: ability of the translation algorithm to adaptively track changes of the features and generate an appropriate output.

#### BCI application

Feature translation generates commands that can control external devices (BCI applications): cursor [57–60] for letter and text selection on the computer screen [44, 45, 61], wheelchair [62, 63], and robotic arm [64, 65]. For BCI restoration problems, the control signals from the BCI application may be transmitted to the brain or other body organs.

## Applications and future of brain–computer interface

In this contemporary society, scientists and engineers have been striving to apply advanced technologies in improving quality of human life [144]. Of the available technologies, BCI has gained considerable attention in medicine for its ability to restore emotional and physical strength of people with missing or damaged body parts. The BCI technology allows physically challenged people to control machines using their thoughts. This advantage gives such people a revealing experience to interact with the external environment and accomplish different activities without dependence from healthy people.

The BCI field is moving fast with a number of promising outcomes that can significantly improve human lives. Researchers require regular updates to address challenges hindering further advancement of the BCI technology. More importantly, given the multidisciplinary nature of brain–computer interface, scientists and engineers should work together to develop new and advanced BCI applications. Recently, the technology has found numerous industrial merits in a range of fields, including mining and education. Combined with fourth industrial revolution, researchers have demonstrated that BCI may accelerate the evolution of robots and neurophysiological discoveries [98, 99, 150]. Other applications of the BCI technology include decoding of thoughts, extension of human memory, telepathy communication, automation and control, intelligence sharing, brain energy harvesting, and optimized (targeted) treatment of damaged body parts.

### Decoding of thoughts

The brain, being a complex human organ, generates and controls our thoughts and other physiological parameters: emotion, touch, breathing, hearing, motor skills, hunger, temperature, memory, and anger. Some parameters, such as anger and changes of breathing rate, may be manifested outside through physical expressions or actions. However, most parameters can only be manifested internally (inside the brain) without the knowledge of other people. The current technologies cannot, for example, predict with an acceptable accuracy the thoughts of an individual. While this internalization of human thoughts—represented as brain signals in a BCI system—may have advantages, some situations may demand us to accurately decode such thoughts. In criminology, for example, policemen would like to understand whether a suspected criminal speaks the truth. Recently, researchers have been investigating how BCI can improve the performance of polygraphs that measure the degree of truth in the arguments from a person (e.g., criminal) [2, 66–68]. Perhaps the promising results in this direction may be achieved by combining BCI and artificial intelligence techniques.

Can the BCI application facilitate translation of human thoughts accurately into a readable text? How can the accuracy of the translated text be measured? Can our imaginations be mapped into real objects, such as pictures and texts printed on a piece of paper? Can events in the dreams be accurately decoded by the BCI system? Can we extend the applications of BCI to develop wearable devices that monitor thoughts or sleeping patterns [69–71]? Can we extract a will directly from the thoughts of a dying person? Can we print physical documents by sending command signals and data from the brain, through the BCI system, to the printer? These interesting questions need further scientific inquiry.

This study envisages that future developments of brain–computer interface will include sophisticated products that can directly map human thoughts into physical objects. We believe that, with the growing trend of BCI, people (especially those with physical disabilities) will drive and control machines (e.g., drones, vehicles, and airplanes) remotely using their thoughts [72]. The advanced developments of BCI may surface critical security and privacy issues, and hence the technology needs to be well-regulated through universal standards [73, 74].

### Extension of human memory

Stephen Hawking theorized the possibility of uploading the human mind into a computer [75]. This philosophical argument, despite its focus on the human mind (consciousness), raises a critical question on whether BCI may be a promising future technology to realize the concept. Specifically, how do we extract memory signals from the brain and decode them for storage into a computer (memory extension)? If successfully implemented, humans will be able to upload their memories into the computer for quicker processing, retrieval, and transmission of information, or for control of external devices.

In the recent developments of brain–computer interface, scientists have generated outstanding results showing that brain signals can be harvested and converted into data reflecting human intended actions [76, 77]. Future studies on BCI may advance these results to investigate how BCI may be used to harvest behaviors and traits from humans for research and scientific study purposes. But this inquiry should be pursued under strict ethical guidelines, a component that has not been well-captured by the BCI researchers.

The sensitive information from the brain, if accurately harvested, may be stored into and retrieved from the external physical memory. Imagining the future of BCI, we envisage that scientists and practitioners may develop portable flash drives (or other variations of physical memories) that may be plugged into the BCI device to extract information from (or introduce information into) the brain. One may question a possible area that may apply the proposed idea. Imagine a counselling psychologist armed with accurate information (obtained through a BCI device) on the behaviors and traits of a person. Evidently, this expert may be expected to provide a well-informed advice and conclusion, giving an appreciable impact to a person being counseled. Achieving this scientific endeavor requires an intensive multidisciplinary research.

### Telepathy communication

Rao et al. demonstrated that BCI, in conjunction with the computer–brain interface (CBI) [78, 79], may allow individuals to communicate without physical interaction or sensory channels [80], a process called telepathy communication. Integration of BCI and CBI forms brain–brain interface that is still in early stages of research and development [81–84]. In future, we expect more work in this direction to expand the applications of telepathy communications in various science and engineering fields. As an example, researchers may investigate how human brains can be interconnected over the Internet of Things (IoT) network to enhance exchange of information and experiences among individuals. While few studies demonstrate the possibility of interfacing BCI and IoT [85–90], linking brains and IoT over the network remains an open-ended challenge that deserves attention of researchers. Furthermore, integration of BCI-IoT and other communication modalities, such as mind–mind interface and mind–machine interface, need further investigation to explore additional capabilities and functionalities on human–machine–human communications. All these technological advancements should, however, be made in parallel with adherence to ethical principles of humanity.

### Automation and control

The promising developments in BCI suggests that the technology may be useful in automation and control industries [91–96]. Currently, BCI has received a significant deal of attention in home automation and control [97]. In this scenario, the technology assists physically challenged people to automate their daily home activities, making it possible for such people live independently. As the technology advances, we expect positive impacts of BCI in the industrial manufacturing processes. In essence, researchers may attempt to investigate the role of BCI in the fourth industrial revolution [98, 99]. For instance, the BCI application may be connected over a secure wireless network to automate processes in the manufacturing industry. Considering sophistication and rapid development in the sensor technology, BCI may be applied in non-contact control and automation industrial systems. This research direction requires intensive investigation to overcome inherent limitations of the BCI technology and ensure seamless interaction with intelligent sensors.

### Intelligence sharing

Can the BCI, in conjunction with the CBI, help to reprogram the brain, hence allowing sharing of intelligence between individuals? Although it may be imagined as a fiction, the fundamental principles of the technology suggest that brains may be reprogrammed artificially. Achieving this milestone, however, requires solid understanding on the nature and functioning of our brains—a stage that has not been reached by the current state of knowledge.

### Brain energy harvesting

The human brain takes only 2% of the body’s mass and, for an average adult in a normal state, consumes 20% of the whole body energy budget to execute its activities [100]. This proportion of energy consumption makes it the third most energy-hungry body organ [101]. We hypothesize that the BCI technology may be combined with other advanced technologies to harvest portion of this enormous amount of energy for powering low-energy external devices. Studies are needed to realize the idea, investigating how much energy can a typical BCI system harvest from the brain.

### Localized brain–computer interface

In BCI, the process of brain signals acquisition is not discriminatory. Virtually, the electrodes acquire all the available signals within the vicinity of its location (under or on the scalp). Consequently, a huge amount of signals and noise are collected for a single intended task (e.g., movement of the artificial leg), making the processing of such signals rather difficult. We can, however, tap the specific signals intended to control a targeted body part by localizing the BCI system. For example, considering a person with speech problems, the BCI system may be placed in an area that directly receives speech control signals from the brain. This advancement may improve the performance of the BCI system and reduce its size.

## Trend of BCI research

In analyzing the trend of BCI research, we, on 26 August 2022, extracted metadata of 25,336 publications from Scopus.^1^ The search string used was “brain computer interface” that, as per the Scopus research rules, includes other similar string variations: brain-machine interface; Brain Computer Interface; Brain-Computer Interfaces; Brain-computer Interface; Brain Machine Interface; Brain-computer Interface (BCI); Brain Computer Interfaces (BCIs); Brain-computer Interfaces; Brain-machine Interface; Brain Computer Interface (BCI); and Brain-Computer Interface. Next, some publications incorrectly classified as related to BCI were omitted. In our extended dataset,^2^ all the extracted metadata were organized into continents, regions, and countries^3^ for analysis. The VOSviewer^4^ served a purpose of organizing and analyzing the bibliographic networks of the investigated BCI publications.

**Fig. 2:**
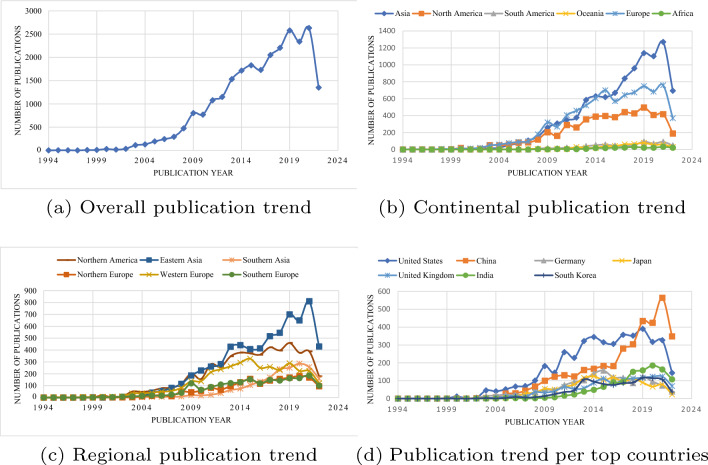
Evolution of brain–computer interface publications. (Data collected from Scopus on 26 August 2022.)

Our analysis reveals that the BCI field has constantly been evolving over the years, with publications ranging from theories and fundamental principals to practical applications. Studies demonstrate that BCI may significantly improve the quality of life for physically challenged people [77, 102]. Given its broad applications in many fields, researchers have invested more time to address practical challenges in BCI systems. Analyzing previous BCI studies, we have observed an exponential growth of the BCI field to date (Fig. 2a). Within a 5-year interval (between 2016 and 2021), for instance, the number of BCI publications increased steadily by approximately 1.5 times. This trend suggests an increasing demand of BCI to the scientific and general community, an indicator calling for a need to conduct advanced BCI research.

Figure 2b, c shows that Asia, specifically the Eastern region, has generated more BCI publications over the years. China demonstrates a steadily growing trend of the publications on brain–computer interface, topping other countries from 2019 onwards (Fig. 2d). This interesting trend may be caused by an increased research funding and support by the China government to undertake advanced research [103, 104]. In the *Made in China 2025* [105] strategy, China has established ambitious plans to become a leading superpower by 2049. The strategy, coupled with a higher population size and an increased number of academic and research institutions, could be a driving factor for China to achieve a remarkable achievement in BCI research.

**Fig. 3:**
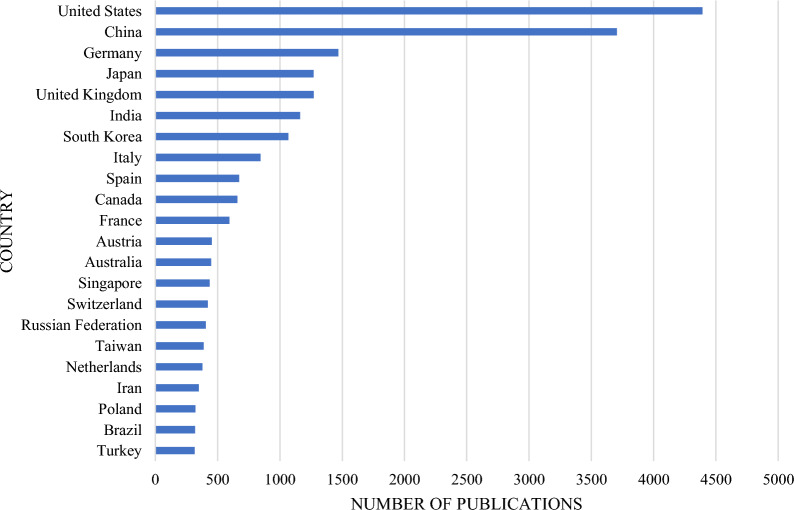
Number of publications on brain–computer interface per country. (Data collected from Scopus on 26 August 2022.)

**Fig. 4:**
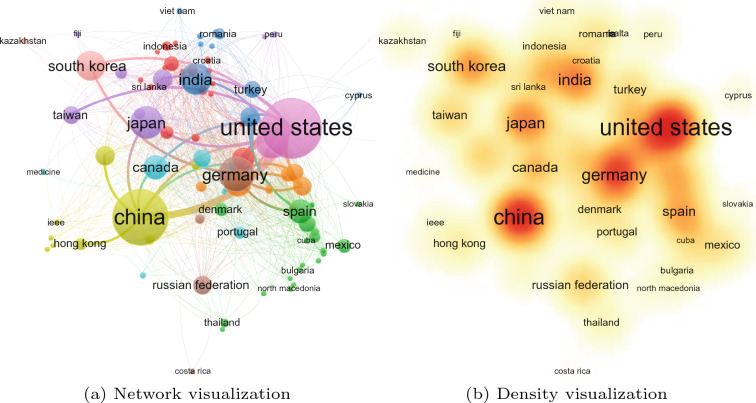
Collaboration network among countries based on publications in brain–computer interface

The United States, however, remains a leading country in terms of the overall number of BCI publications (Fig. 3). Given the higher technological and economical muscle of the United States, this observation would be expected. Perhaps an intriguing question for future inquiry would be on why the number of BCI publications for this country started to decline from 2019 onwards. One way that the United States may improve the trend of BCI publications is to promote co-authorship with Chinese universities and research institutions (Fig. 4).

**Fig. 5:**
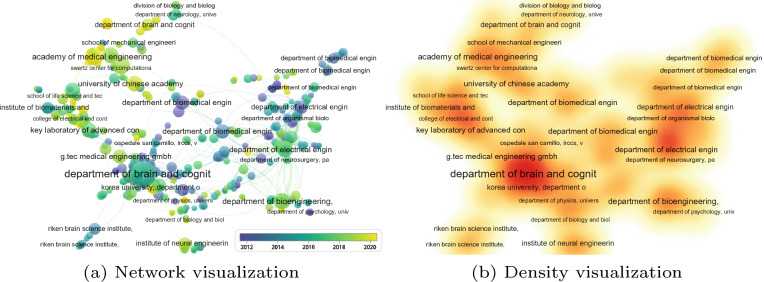
Collaboration network of organizations supporting research on brain–computer interface. (Data collected from Scopus on 26 August 2022.)

Figures 4 and 5 show five countries with higher volume of BCI publications: United States, China, Germany, Japan, and India. Authors from these countries collaborate to foster the development of BCI research. Given the value of BCI technology in human socio-economic development, we recommend the efforts to be adapted in other countries, specifically those in the global south. Institutions from low-income economies, as defined by the World Bank, should be empowered to conduct advanced BCI research with a focus on addressing the third sustainable development goal, “good health and well-being”.

Africa lags behind in BCI research (Fig. 2b), generating only 0.95% of all the BCI publications globally. This small proportion may be attributed to insufficient funding for supporting and advancing BCI research (Fig. 5). Funding organizations may need to observe Africa as a potential continent for BCI research. With an estimated population of 1.426 billion people by 2022^5^—approximately three times that of Europe^6^—and with more than 2,000 universities and institutions,^7^ Africa can significantly contribute in BCI research. The methods and results from studies on BCI can improve the quality of life for millions of Africans. According to statistics from the United Nations, more than 80 million people in Africa are disabled, including those with severe mental health conditions and physical impairments that may be beneficiaries from BCI results. Therefore, supported by funding organizations and governments, African researchers and innovators should exploit the capabilities of BCI technology to address the existing practical challenges in Africa. Another possible reason causing low number of BCI publications in Africa could be the inadequate level of technology to undertake BCI research that requires advanced equipment and complex infrastructure. Collaboration with the developed world, especially China and United States, in undertaking BCI research may be an effective and a feasible strategy for Africa to achieve the desirable output in BCI research.

Generally, the BCI research opens up several interesting problems that demand attention within the scholarly community. Our study discovered that countries address the BCI problem differently depending upon their local contexts. For example, while BCI studies from developed countries focus on the industrial applications of the technology, those from developing countries mostly deal with how the technology contributes in improving life quality of humans (e.g., increasing life expectancy). United States and China, which have shown significant advances in BCI research, provide promising prospects of BCI in the fourth industrial revolution [98, 99] with, however, a serious concern of the potential threats that the technology may impose if misused. These countries have, in fact, practically applied BCI in the real-world to advance humanity. Critically analyzing metadata of the 25,336 reviewed articles, we observed sophisticated BCI research laboratories^8^, ^9^, ^10^ that generates results with positive practical impacts. Developing countries, such as those in Africa, lack a support infrastructure for BCI research. Therefore, it may be relatively challenging in these countries to comprehensively explore competitive advantages of the BCI technology.

## Challenges and potential threats of brain–computer interface

The BCI technology, despite its broad applications, poses threats to humans that need to be addressed. As we strive to make the technology friendly and useful, researchers should develop BCI applications that resonate with the standard principles of humanity. In essence, a better technology should enhance our lives while considering human factors, including convenience, ease-of-use, privacy, security, and safety [106–108]. Before adopting the BCI technology for use by the community, researchers and practitioners are obliged to engage users and ensure that the technology has passed predefined quality standards.

### Privacy

In the article by Luigi Bianchi,^11^ the author informs lack of specific standards that govern development of BCI applications. This challenge, as noted by Takabi et al. [109], has resulted in BCI applications with unrestricted access to brain signals. The authors’ results show that these applications may, as a consequence, extract sensitive information from users without their knowledge. As an attempt to address privacy concerns, standards should be established to define acquisition methods, access control protocols, and encryption techniques, among other attributes. Klein and Ojemann suggest that the privacy concerns and other threats may be addressed through adherence to best practices when developing BCI systems and incorporating such concerns into the informed consent protocols [110].

**Fig. 6:**
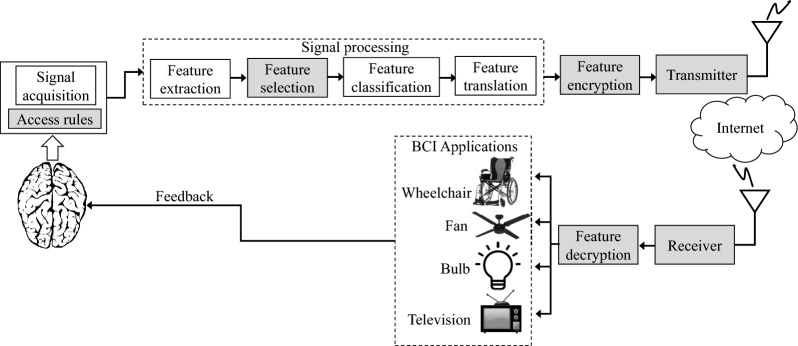
Brain–computer interface (BCI) system with encryption and decryption components for enhancing privacy

In this work, we have hypothesized a functional model of the BCI system that accounts for privacy and security issues (Fig. 6). This model, which extends the work of Mason and Birch [111], contains components that may prevent unauthorized access of sensitive personal information without the user’s awareness. Recalling Fig. 6, before acquisition of brain signals, the BCI system engages the user with predefined access rules to ensure high integrity and privacy of information. In the signal processing block, a component “Feature selection” retains quality features intended for classification and translation. Next, for BCI applications linked with networked devices over the Internet, we propose encryption of the translated features (control commands) before transmission. This process prevents attackers from altering the control commands, a consequence that may threaten the user’s safety. Other advanced technologies, including blockchain [112], may also be used to prevent unauthorized access of the control commands by the attackers. Lastly, the model contains a feature decryption block that decodes the encrypted control commands for use by the BCI applications.

### Security

The field of BCI has made a significant progress in the development of medical applications and products to improve the patients’ quality of life (e.g., restoration of damaged sight or hearing) [113]. However, given the increasing demand for BCI-internet communications, security concerns have emerged [114–116]. The advancement of brain–computer interface creates opportunities for cyber attackers to intervene in the normal operations of the BCI application [117]. The attackers may alter commands derived from the feature translation component (Fig. 1) and cause adverse effects to the target subject. Therefore, researchers should investigate security threats and vulnerable BCI components that can be easily attacked, then find robust solutions.

### Safety

Safety concerns can generally be observed in invasive BCI types. Because of being implanted into the brain tissue, invasive BCI can damage nerve cells and blood vessels, hence increasing the risk of infection.^12^ Additionally, the natural defence system of the body may reject the implant, treating it as a foreign entity (biocompatibility concern). Another safety concern of invasive BCI is the possible formation of scar tissue after surgery, a consequence that may gradually degrade the quality of the acquired brain signals. Addressing this challenge requires a comprehensive knowledge on how the human body works and interacts with foreign matters. The knowledge should be used by BCI scientists and engineers to develop safe and quality BCI applications. This knowledge should, in addition, equip neurosurgeons with more accurate information on specific brain regions to implant BCI electrodes.

### Ethical, legal, and social concerns

The BCI research raises a number of ethical, legal, and social concerns on privacy, security, safety, accountability, and accessibility [118]. The society would prefer the BCI technology that addresses their questions. For example, should people be concerned by privacy and security of the BCI applications? Does the technology guarantee safety? Does the society get equal access to the technology? In a situation of negative technological or technical impacts, who will be accountable and what are the legal implications? These questions require careful considerations and further research before administering this technology to the society.

### Convenience and flexibility

Most BCI applications require calibration data to reverse undesirable changes caused by neural plasticity or micromovements of the electrode arrays [77]. This necessity calls for frequent decoder retraining, an inconvenient and time-consuming process that unnecessarily burdens the user. Willett et al. [77] highlight the challenge in their seminal work on brain-to-text communication through handwriting. Despite the promising performance achieved by the authors’ model, daily decoder retraining was unavoidable. Future studies may investigate more effective techniques for decoder training without physically engaging the user. In essence, the BCI application should operate adaptively with respect to the stochastic changes in the neural activities of the brain. Automatic self-calibration approaches may be employed to update operation of the BCI application accordingly, hence promoting convenience and flexibility.

### Multidisciplinarity

**Fig. 7:**
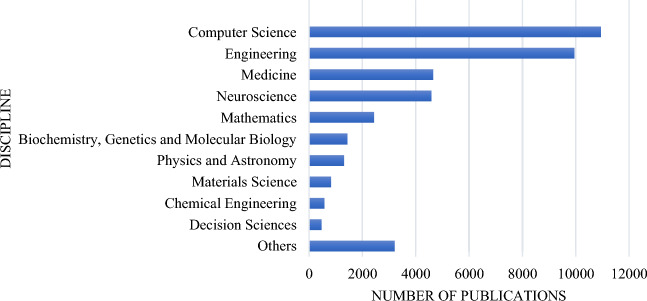
Number of brain–computer interface publications per discipline

The BCI field involves multiple disciplines that should be linked to establish advanced principles and more effective BCI applications. In our analysis from Scopus, we observed that some important disciplines have not been adequately engaged in the BCI research (Fig. 7). For example, only 1% of the BCI-related publications originate from psychology, a discipline dealing with study of human mind and behavior. Psychology, when combined with other disciplines, may provide a milestone to develop even better and practical BCI systems that can revolutionize humanity positively. Establishing research teams from varied disciplines may require strategic plans and funding, but such multidisciplinary teams are important to fully harness the BCI promising capabilities.

### Big data

The brain stores an enormous amount of information serving different human tasks. In addition, this central body organ generates a vast amount of electrical signals that control, monitor, and regulate human activities. Evidently, BCI raises a big data problem that needs sophisticated techniques to address. Unfortunately, because of insufficient knowledge on the brain working principles, BCI researchers may not have collected and utilized all the brain data and signals. Researchers need to understand key neurological features, including neuroplasticity that flexibly allows re-organization of neurons in learning or injury recovery [119]. In non-invasive BCI, researchers should determine resolution of the electrode network on the scalp for optimal collection of brain signals. Similarly, invasive BCI requires electrodes optimally positioned under the scalp.

### Availability of participants for clinical trials

BCI, being an emerging and a relatively new technology, offers promising opportunities to several disadvantaged groups. Most people, especially those from developing countries, are unaware of the merits and demerits of the technology as evidenced from a smaller number of BCI publications collected from such countries (Fig. 2b). Therefore, engaging an acceptable number of people in testing the BCI medical products may be relatively challenging.

Following ethical guidelines, people should express their consent to accept, adopt and use the BCI technology. In this work, we noted limited attempts to start clinical trials of BCI devices. On 28 July 2021, Synchron became the first BCI company to receive approval from the United States Food and Drug Administration for conducting (investigational device exemption) clinical trial of a permanently implanted device, Stentrode^13^ [120]. Other initiatives for clinical trials of BCI products can be observed at the University of Pittsburgh^14^ (sensorimotor microelectrode brain–machine interface) and the United States National Library of Medicine^15^ (e.g., BrainGate2^16^ [121] and BCI device from the University of Grenoble [122, 123]). Morinière et al. introduced a dual-arm exoskeleton for evaluating BCI products in clinical trials [124]. Despite these initiatives, including those from startups and companies, the number of participants involved in the clinical trials seems insufficient for generalization across the global community. We recommend diversification and increased number of participants for clinical trials from different countries, considering cultural and traditional values. Furthermore, studies may be needed to understand acceptance of the BCI technology to the society. In this work, we located a few studies that attempt to determine human behavioral factors towards acceptance of BCI devices [125, 126]. Our recommendation is that, despite the advantages that this technology provides, the development of such devices should consider the factors.

### Standardization and approval by regulatory authorities

**Fig. 8:**
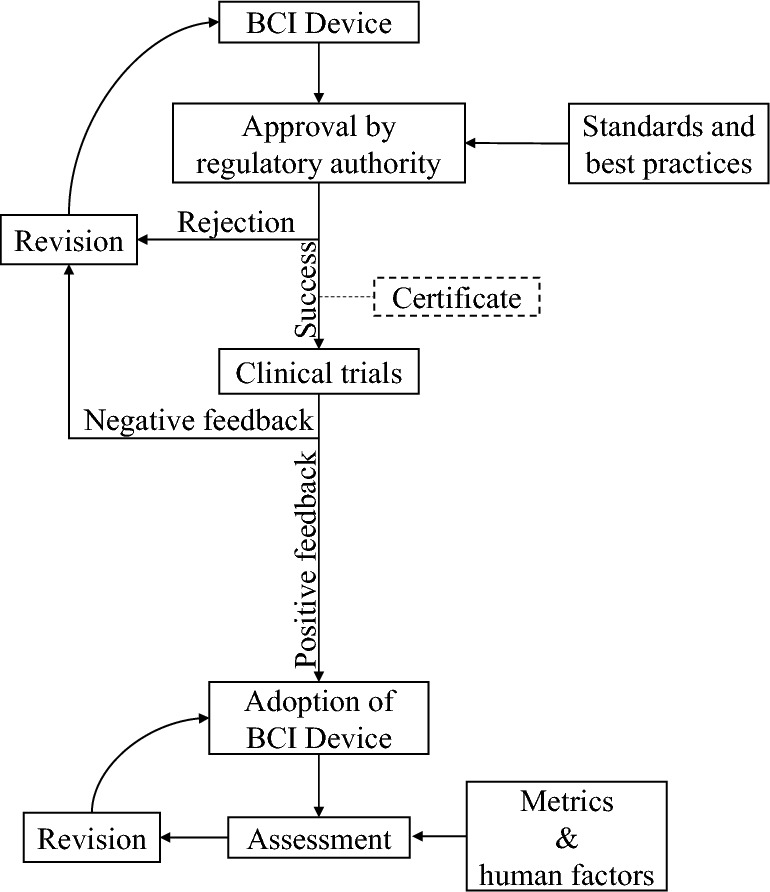
Proposed procedures for practical administration of brain–computer interface devices

We have witnessed an increasing number of initiatives to develop BCI devices with advanced features^17^, ^18^ [119, 127]. Startups and companies have been developing commercial BCI devices for use by the society. Our study found ongoing efforts for developing universal standards governing neurotechnologies for BCI devices.^19^ These efforts should be accelerated to match with the increased commercial demands of the BCI devices. Currently, people may raise concerns on the practical suitability of the BCI technology with respect to general quality and ethical guidelines. In addition, guided by the best practices for developing and administering medical devices, information on clinical trials for the commercially viable BCI devices remains unclear. We could locate from public medical databases only a few clinical trials with limited number of participants. Considering the delicacy and possible long-term impact of BCI technology to humans, approval procedures from respective regulatory authorities seem necessary before commercialization of BCI devices (Fig. 8). This necessity, however, introduces another challenge that some developing countries may be inadequately equipped with advanced facilities and expertise to test and approve BCI devices.

### Battery lifetime

Implantable BCIs require materials that can sustainably operate over longer periods of time, preferably decades, without deterioration [119, 128–130]. The warm aqueous nature of our brains, however, affects the power-retention capability of the implants. Water (cerebrospinal fluid), being a powerful solvent, gradually corrodes the insulating materials of the electrodes. Over time, short circuits may be created, increasing crosstalks between electrodes. This challenge reduces battery lifetime and limits the amount of signals collected by electrodes. Researchers need to study different insulating materials to understand how they interact with the brain relative to the BCIs battery lifetime. In addition, computationally efficient algorithms should be developed to ensure optimum utilization of battery power. Even more importantly, alternative energy sources (e.g., micromovements inside the brain) for powering implantable BCIs should be investigated.

### Affordability and portability

Commercially available BCI devices can hardly be afforded by the general public because of their prohibitively high costs [131–134], perhaps due to their sophistication and construction materials. Also, the current BCI systems are complex and bulkier, making them suitable only in laboratory and industrial settings. Researchers should develop cost-effective and portable BCI systems for ordinary people, potential users of the technology. This solution will be more useful for people in developing countries.

## Conclusion

In this study, insights have been given on the perspective of the brain–computer interface. Inspired by its benefits, the society needs to seize the available opportunities that the technology advocates. To maximize the benefits and increase usability of the BCI technology across the society, researchers and scientists should address the potential threats of the technology highlighted in our work. We can fully exploit the benefits and capabilities of the technology through multidisciplinary efforts to address limitations of the current BCI systems.

In view of the BCI components, five possible research directions can be taken: cognitive psychology, medicine, biomedical electronics, signal processing, and engineering. These directions necessitate multidisciplinary research where researchers work closely to address the BCI sub-challenges. Psychologists and medical doctors should provide the fundamental working principle of the brain; scientists should develop effective signal acquisition devices along with algorithms for processing brain signals (extraction, classification, and translation of features); and engineers should develop physical BCI applications and evaluate their performance based on the predefined standards.

We assert that the BCI field has many research opportunities that have not been explored. From all the reviewed literature, an observation was made that the existing challenges in brain–computer interface have received little attention. The research community is recommended to address the challenges and extend the capabilities that BCI offers, including development of BCI-Internet and BCI-CBI communication devices. In addition, researchers may explore how mind–body intervention methods, such as hypnotherapy, can improve BCI systems [135–137]. In whatever situation of development, however, the primary goal of BCI should be to advance humanity by improving the quality of people’s lives.

Notwithstanding the promising capabilities and merits of BCI, a significant number of challenges and threats have not been adequately addressed. In addition, the current number of participants in the clinical trials seems low and undiversified, making generalization of the results questionable. Furthermore, global standards should be established to develop safe and quality BCI products with threats significantly minimized. In this regard, although BCI unlocks our future for well-being, this emerging technology requires intensive research, including many clinical trials, for practical applications. With the existing challenges and threats unsatisfactorily addressed, the technology may not be ready for consumption by the society. This conclusion is partly supported by a few other studies [138–144] and scholarly communications.^20^

Our future work will be focused on addressing some threats originating from the middle BCI component, signal processing. Using the publicly available dataset^21^, ^22^ [145–149], we will develop computationally inexpensive algorithms for encrypting, extracting, classifying, and translating features from the brain. Measures of accuracy will be established to ensure that the developed algorithms give computer commands that accurately emulate users’ actions. Note that there has been no universally acceptable standards for measuring the accuracy of BCI applications, and we will attempt to narrow this research gap.

## Data Availability

The authors declare that the data supporting the findings of this study are available within the article and its supplementary information files.

## Footnotes

1 https://www.scopus.com.

2 https://drive.google.com/drive/u/0/folders/1vcamDdm4oNaPtm5ktWkTaiT6LMmOEE-h.

3 Link of continents/countries/regions:https://statisticstimes.com/geography/countries-by-continents.php.

4 https://www.vosviewer.com/.

5 https://worldpopulationreview.com/continents/africa-population.

6 https://ec.europa.eu/eurostat/web/products-eurostat-news/-/ddn-20220711-1.

7 https://www.webometrics.info/en/Africa?page=20.

8 https://about.bci-lab.info/.

9 https://www.cmu.edu/bme/helab/Research/BCI/index.html.

10 https://www.etsu.edu/cas/psychology/bcilab/.

11 https://lifesciences.ieee.org/lifesciences-newsletter/2019/april-2019/on-brain-computer-interface-standards/.

12 UK Parliament POST, Brain-Computer Interface; POSTNOTE: Number 614 January 2020.

13 https://synchron.com/.

14 https://www.rnel.pitt.edu/.

15 https://clinicaltrials.gov/.

16 https://www.braingate.org/.

17 https://www.ai-startups.org/top/brain_computer_interface/.

18 https://tracxn.com/explore/Brain-Computer-Interface-Startups-in-United-States.

19 https://standards.ieee.org/industry-connections/neurotechnologies-for-brain-machine-interfacing/.

20 https://www.technologynetworks.com/neuroscience/blog/exploring-the-ethical-challenges-of-brain-computer-interface-technology-363367.

21 http://www.bbci.de/competition/iii/.

22 http://bnci-horizon-2020.eu/database/data-sets.
